# The Cause of the Increase of Diseases of Women, and Their Prevention

**Published:** 1894-09

**Authors:** Geo. W. Christian

**Affiliations:** Houston, Tex.


### Southern Medical Record.

A MONTHLY JOURNAL OF MEDICINE AND SURGERY.

VOL. XXIV. ATLANTA, GA., SEPTEMBER, 1894. No. 9.

### Original Articles.

### THE CAUSE OF THE INCREASE OF DISEASES OF WOMEN, AND THEIR PREVENTION.

GEO. W. CHRISTIAN, M.D., HOUSTON, TEX.

We have no means at hand that will enable us to compare the number and percentage of sick women of to-day with those of twenty or a thousand years ago, but that there has been and is an increase is, I believe, a universally admitted fact.

I shall treat the subject under the head of "Heredity, and Acquired Causes," believing that both play very important parts in the development of this as in all other classes of diseases.

That the laity appreciate the facts of heredity as applied to the animal and vegetable kingdom, is evidenced by the fact that they select the best seed for planting, the best plants for transplanting, the most promising chicken, pig, sheep, goat, cow, horse, dog, or ass to propagate from, selecting the best, not from one sex alone, but from both male and female. The fact that so little attention has been paid to the laws of heridity in the human family, when it is so well understood, and so universally practiced and applied to the vegetable and animal kingdom with such satisfactory results, has been to me a matter of much surprise.

Why is it that a man, the noblest work of God, the masterpiece of his creating fiat—the acme, and last of all his glorious works, will refuse his cock to an inferior hen, and his ass to a puny jennet, but will lend himself a willing victim to the commonest of womankind, struggle ingloriously on to plant his seed in a soil that can but half nurture to maturity, and that will insure moral degradation, physical torture and mental aberration in his progeny forever. And woman, God bless her, with all her purity, goodness and beauty, will join her heart and hand, prostitute her proud and lovely form to mother children for a half generate, poorly conceived, illy begotten and dwarfed son of Adam. Would you doubt these influences? Then go with me to the florist and trace the beautiful rose, with its profusion of fragrant petals, scintillation of beautiful colors, with all its varied sizes, shapes and charms of fragrance and color, back to the homely two or three petaled progenitors, and so of any other of his flowery kingdom laden with beauty, sweetness and perfection of form. Trace back the fruits with their rich stores of sweet and palatable juices, with their proud forms and lovely shapes, to their small, juiceless and unpalatable parents, and do not stop with the fruit and flowers, but take the whole vegetable kingdom from the alga to the oak, and see what wonderful results have been accomplished by selection of parent stem, proper cultivation and adaptation to soil and climate. In the animal kingdom like results have been reached by close attention and due observance of these same laws, the horse has been brought well-nigh to perfection and made for speed or draught, light or heavy weight, long or short distance, to meet the wants or gratify the fancy of man; the cow has been brought to meat or milk, to bone and muscle, to meet the demands for food or bearers of burdens as we will; the sheep to produce wool or mutton, or both, as we choose. In fact, we see the results of the application of this law in all our domestic animals. They have been made near perfection for the purpose for which we choose to use them, whether goat or pig, fowl or ass; these magnificent results have been the rewards of patient, thoughtful, intelligent application of natural laws, well understood by us, applied to all vitalized matter.

Not only should we have a healthy plant or animal to begin with, but the food and surroundings should be in perfect accord with the demands of each particular plant or animal. Not that perfection cannot be obtained without this (for that has already been claimed), but the better the parent, the nearer perfection is the offspring, or in other words, the shortest time will be necessary to attain it.

It is to be hoped that the human family will avail themselves in the near future of the blessings that would surely result if these laws were applied in our matrimonial alliances. At present, however, we will have to take the girl baby as she comes to us, whether shapeless in form, pure in blood, or tainted and imperfect, and make the best we can of her. Realizing that thirty or more chemical elements enter into her composition, let us study the manner in which they enter, and the conditions under which they are appropriated to meet the ever-changing demands of waste and repair. Enter the body as they must through the lungs and stomach, our first effort should be to see that the substances entering contain the proper elements only and are in quantity and quality suited to the demands of each particular individual, for each will demand changes at times as to quantity and quality to meet the demands, as will result from different forms and amount of labors of the individual. Then the lungs and stomach, to insure a perfect, vigorous and healthy development, must be able to appropriate the elements furnished them, and the nearer these organs approach to health, the better will be the results. Then the first efforts should be to keep the lungs and stomach healthy, and afterwards to furnish pure air and proper, wholesome food.

We appreciate how important a healthy stomach is, when we look over the annual deaths and see how many are put down to gastritis, enteritis and colitis, to say nothing of marasmus and other wasting diseases which come from imperfect digestion, the stomach or intestinal canal being at fault (and when I say stomach in this connection, I wish to be understood as meaning the whole digestive tract). Hundreds and thousands who live through these acute troubles carry their digestive capacity with them to the grave, many of them never having known anything but sighs, aches and pains during their short and miserable lives.

Next to over and under and improper feeding come foul air, overcrowding, improper dressing, with neglect of sunshine and exercise of the muscular system, with minds burdened with too numerous and long lessons. Thus the girl reaches the first approach of puberty, with its strong demands upon her, with an imperfect development, dwarfed body, impoverished blood and tired nerves, wholly unfit to assume the extra strain levied upon her, and too hurried upon her by the numerous errors of education and social influences.

Our educators do not seem to appreciate, as they should, the importance of daily outdoor exercise, and hence send our girls away to fashionable boarding schools, which generally mean pale faces and broken health. Add to these errors late nights, long courtships and marriages before maturity of the sexual system, with inordinate sexual indulgence, and we have cause enough to account for the numerous aches and pains so common to American womanhood.

We have just now reached the period of motherhood, with all of the ills that follow childbearing and nursing in healthy normal women, with a puny, half-developed girl to assume the trying strain incidental to married life. Ruptures and tears will occur from malpresentations and monster children in the hands of competent men in normal women, but they must be more frequent in the poorly developed mothers and under the management of midwives and incompetent men.

Miscarriages, which seem to be the order of the day, are responsible for much of the suffering of women. To this and to gonorrhea in the husband may be traced the large majority of exciting causes of the diseases of women. These two factors are the ones which gives most of the work to the abdominal surgeon. Neglect, improper treatment with unclean instruments, the introduction of ill-fitting pessaries, claim a liberal share of victims. Efforts to thwart conception, such as condoms, washes and withdrawals, all have their share of responsibility. Some of the most pitiable invalidism that has come under my observation has been the result of these sins.

The constitutional causes that produce a tendency to diseases peculiar to women are syphilis, scrofula, and in fact all vicious habits and tendencies in the parents, and these causes, too, may be considered with the acquired as the predisposing factors; miscarriages, childbearing, excessive sexual indulgence, uncleanliness, taking cold, overwork, strains, etc., as the exciting. It is generally understood that one-half of the diseases peculiar to women develop as a result of childbearing, and from that standpoint, it might appear that the profession of medicine then was responsible; but I hold that did not the predisposing causes exist, we would not have so much trouble in the obstetric chamber. The doctor is too late when he confronts the labor to give size, strength and form to the sexual organs. This should have been done all along the line, in the first place by judicious marriage, the latter by proper exercise and adaptation to surroundings with a variety of plain and wholesome foods

The prevention of the diseases of women has already been overshadowed by what has been said. If we can do nothing to impress the facts and importance of heredity upon the candidates for matrimony, we may be able to contribute somewhat to the strength of the race by urging upon the people the necessities of better sanitation, exercise adapted to the strength and needs of the individuals, together with proper bathing, foods, etc. By prompt and intelligent action on our part, much comfort may be added to our patients and much distress and invalidism avoided. We can by proper restrictions of the practice cut off the incompetents (but we must have the people with us to do this), and save womanhood such sufferings as comes from malpractice, ignorance, meddlesomeness, etc. The other blessings derived from a perfect form only, with every organ perfect, from the sight to the sense of sexual touch, will follow in due time.

To sum up, the hereditary causes of diseases of women are, first—syphilis, scrofula and faulty parentage, with inherited weakness. The acquired causes are, first—improper food and stuffing in infancy; second—the continuance of the influences during childhood; and, third—the overcrowding through school, with neglect of exercise, recreation, sunlight, bathing, etc.; fourth—early marriage and sexual excesses; fifth—gonorrhea and miscarriage; sixth—want of cleanliness in women and incompetency and meddlesomeness of physicians, midwives and officious friends; seventh—late nights, exposure to cold, improper dress, with tight corsets, thin shoes and high heels.

